# Microfluidics delivery of DARPP-32 into HeLa cells maintains viability for in-cell NMR spectroscopy

**DOI:** 10.1038/s42003-022-03412-x

**Published:** 2022-05-12

**Authors:** Nicholas Sciolino, Anna Liu, Leonard Breindel, David S. Burz, Todd Sulchek, Alexander Shekhtman

**Affiliations:** 1 University at Albany, Department of Chemistry, Albany, NY 12222 USA; 2 Georgia Tech, School of Mechanical Engineering, Atlanta, GA 30332 USA

**Keywords:** Solution-state NMR, Intracellular signalling peptides and proteins, Molecular neuroscience, Solution-state NMR

## Abstract

High-resolution structural studies of proteins and protein complexes in a native eukaryotic environment present a challenge to structural biology. In-cell NMR can characterize atomic resolution structures but requires high concentrations of labeled proteins in intact cells. Most exogenous delivery techniques are limited to specific cell types or are too destructive to preserve cellular physiology. The feasibility of microfluidics transfection or volume exchange for convective transfer, VECT, as a means to deliver labeled target proteins to HeLa cells for in-cell NMR experiments is demonstrated. VECT delivery does not require optimization or impede cell viability; cells are immediately available for long-term eukaryotic in-cell NMR experiments. In-cell NMR-based drug screening using VECT was demonstrated by collecting spectra of the sensor molecule DARPP32, in response to exogenous administration of Forskolin.

## Abstract (web summary)

The microfluidic technique of cell volume exchange for convective transfer, VECT can be used to deliver the DARPP-32 protein into cells for in-cell NMR experiments.

## Introduction

The study of protein structure under physiological conditions is the next frontier of structural biology^1^. The intracellular environment is extremely dense and heterogeneous providing both specific interactions that result in high-affinity protein complexes and omnipresent weak interactions that can influence protein structure and activity^2–5^. The lack of bulk water changes the physicochemical properties of proteins and affects the strength of hydrophobic and electrostatic interactions that drive protein complexation^6,7^. In-cell NMR provides a means to observe the atomic resolution structure of target proteins in mammalian cells^8–13^.

Target proteins labeled with NMR active nuclei ^13^C and ^15^N are easily distinguished from the rest of cellular proteome^13^ and can be detected at concentrations as low as 5–10 μM. A common method for introducing labeled target proteins to cells is by overexpression in a labeled medium^11^. However, because large protein complexes are invisible by solution NMR^14^; there is a need to deuterate proteins to observe in-cell NMR spectra^12^. This requirement limits protein overexpression, particularly in mammalian cells, which do not grow in perdeuterated medium. Exogenous delivery of target proteins using techniques such as microinjection^8,15^, cell-penetrating peptides^16^, creation of pores^10^ and electroporation^17,18^; limit cell viability^19^ and may perturb the physiological state of the cells^20,21^ by impeding homeostasis and cell growth^20^.

The ability of cells to rapidly exchange fluid with their surroundings in response to ultrafast mechanical compressions presents a robust method to deliver large extracellular molecules and particles into cells^22–24^. The microfluidic technique of cell volume exchange for the convective transfer, VECT, has been used to deliver molecules intracellularly from particles suspended in extracellular fluid. The critical advantage of VECT over pore-forming techniques for protein delivery is that VECT delivers proteins without creating significant prolonged cellular stress^25,26^. Microfluidic delivery does not destroy the nuclear membrane and protein is delivered only into physiologically relevant compartments^22^.

The effectiveness of microfluidics-based delivery of target proteins into HeLa cells was tested. The viability of cells transfected by using electroporation and VECT was compared and the efficacy of in-cell NMR experiments utilizing a VECT-delivered target protein, DARPP-32, was demonstrated.

## Results

### VECT delivery of target protein promotes high cell viability

Membrane disruption methods are used to deliver biological target molecules intracellularly for in-cell NMR spectroscopy. In-cell NMR requires a long time, ≥3 h, to collect spectra during which cells die, lyse, and leak. Among the most commonly employed delivery techniques is electroporation, however, electroporated cells exhibit damage to membranes, mitochondria, protein, and DNA, decreases in ATP levels as well as increases in reactive oxygen species, ROS, and intracellular Ca^2+^ concentrations, all of which can lead to cell death^27,28^. Thus effective use of electroporation requires optimization of a number of parameters including voltage, cuvette gap size, shape, length and number of pulses, cell size and concentration, buffer and temperature, to strike a balance between transfection efficiency and cell death. This can be particularly inconvenient when the target protein is expensive to prepare or can only be purified in small quantities. The use of a bioreactor helps maintain cell viability but electroporated cells still have to recover from damage. VECT delivery, on the other hand, results in high cell viability and does not require extensive optimization; the basic procedure being applicable to most cell types and target proteins.

**Fig. 1:**
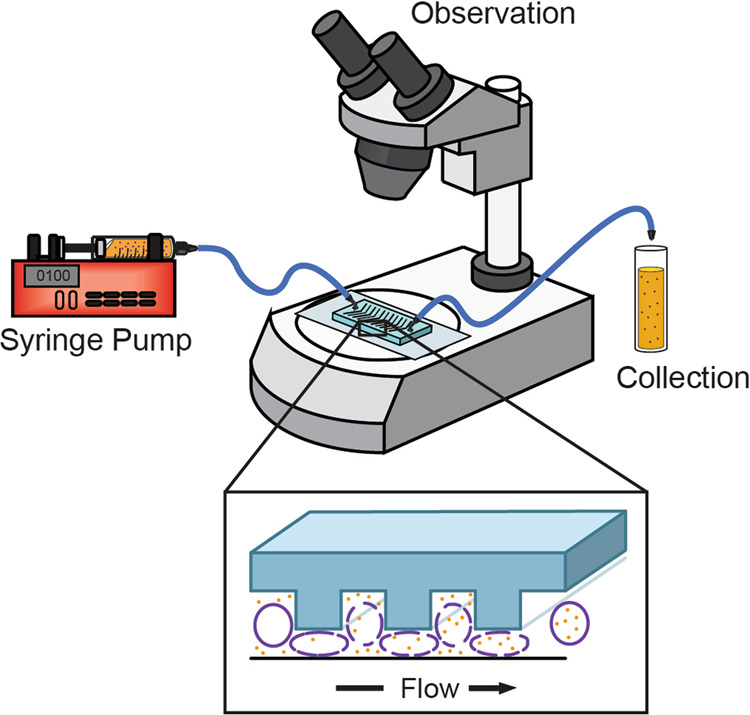
Experimental setup for cell volume exchange for the convective transfer, VECT. A syringe pump delivers the target protein into HeLa cells as it passes through the microfluidic device.

The experimental setup for VECT delivery of proteins into cells is shown in Fig. 1. Microliter to milliliter volumes of cells are flowed through channels ranging from tens to hundreds of micrometers in dimension. Rapid mechanical deformations cause transient cell volume exchange that facilitates the convective transfer of extracellular material into the cell. Many biological macromolecules, such as dextrans, DNA, protein, and oligomers, have been successfully transfected into a range of cell types, including HEK293 and K562 cells, primary neurons, and fibroblasts, neuron-like N1E-115 cells, dendritic cells, blood immune cells, and embryonic stem cells^29^. Previous experiments showed that, as in electroporation, the amount of protein delivered into the cytosol is linearly proportional to the concentration of extracellular protein^22,30^. Successful delivery of protein resulted in minimal, ~10%, rupture of the nuclear envelope, <5% loss of material to the cytosol, and ~2% loss of the cytosolic content during transfection^22^.

Green fluorescent protein, GFP (27 kDa), was used to quantify and compare electroporation and microfluidic protein delivery to HeLa cells. The intrinsic fluorescence of GFP facilitated imaging to assess target protein delivery and cell morphology following transfection. The concentration of extracellular GFP used for both transfection methods was 300 μM. The electroporation pulse program was the same as previously used by our group and others to electroporate HeLa cells for in-cell NMR spectroscopy^12,17,30^. Analysis of lysed cells indicated intracellular GFP concentrations of 20 ± 10 μM and 5 ± 2.5 μM from electroporation- and VECT-delivery, respectively.

**Fig. 2:**
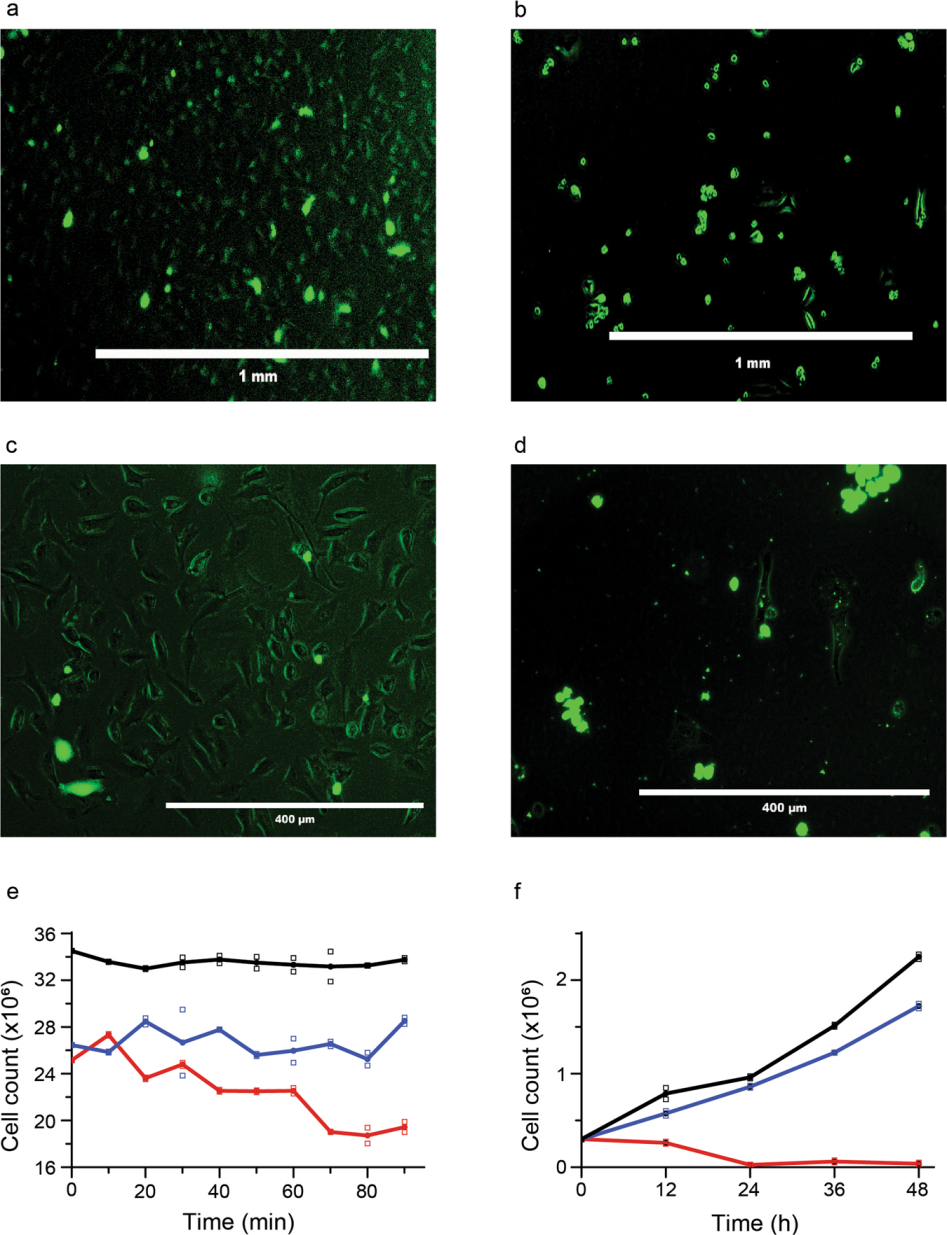
VECT versus electroporation delivery of GFP to HeLa cells. **a**, **c** VECT delivery. **b**, **d** Electroporation delivery. **e** Short-term HeLa cell viability following transfection. **f** Long-term HeLa cell viability following transfection. Non-transfected (control) cells (black), cells with VECT-delivered protein (blue), and cells with electroporated-delivered protein (red). Data were reported as a mean based on two experiments.

VECT-transfected cells exhibited normal morphology (Fig. 2a, c) whereas electroporation-transfected cells were rounder, aggregated, and displayed more concentrated GFP signals (Fig. 2b, d). Cell attrition was assessed in the 90 min window immediately following transfection (Fig. 2e). Electroporated cells exhibited a steady decline in viability resulting in an ~25% reduction compared to VECT, which increased by ~10% over the same period. Long-term recovery showed that VECT-transfected HeLa cells were capable of exponential growth comparable to that of non-transfected cells over a 48 h period whereas the electroporated cells dropped below seeding density in 12 h, and were unable to demonstrate exponential growth by 24 h (Fig. 2f). The viability of electroporated cells after 12 h was 85–90%, comparable to the 75–85% observed by Theillet et al.^18^, i.e., 15–25% dead cells after >13 h. Overall, the higher attrition rates of electroporated versus VECT-transfected cells were consistent with the idea that the electroporated cells were more extensively damaged^19^.

### VECT-delivery of DARPP32 to HeLa cells

Dopamine and cyclic adenosine 3, 5′-monophosphate-regulated phosphoprotein, DARPP-32, is a 32 kD sensor protein found in dopamine-rich areas of the brain that is extremely sensitive to cell physiology^31,32^. Functional studies highlighted the role of the N-terminal region of DARPP-32 as a sensor of cell surface receptors^33,34^. To investigate its structure in live cells, a C-terminally truncated DARPP-32_1-122_ construct was used^35–37^. In vitro characterization showed that DARPP-32_1-122_ is an intrinsically disordered protein, IDP, and contains a partially folded short helix between amino acids 22 and 29^35^. The high signal-to-noise ratio afforded by IDPs, relatively well dispersed ^1^H-^15^N correlation NMR spectrum^35–37^, and high, 30 µM, physiological intracellular concentration makes DARPP-32 an attractive target for in-cell NMR analysis^33,38^.

**Fig. 3:**
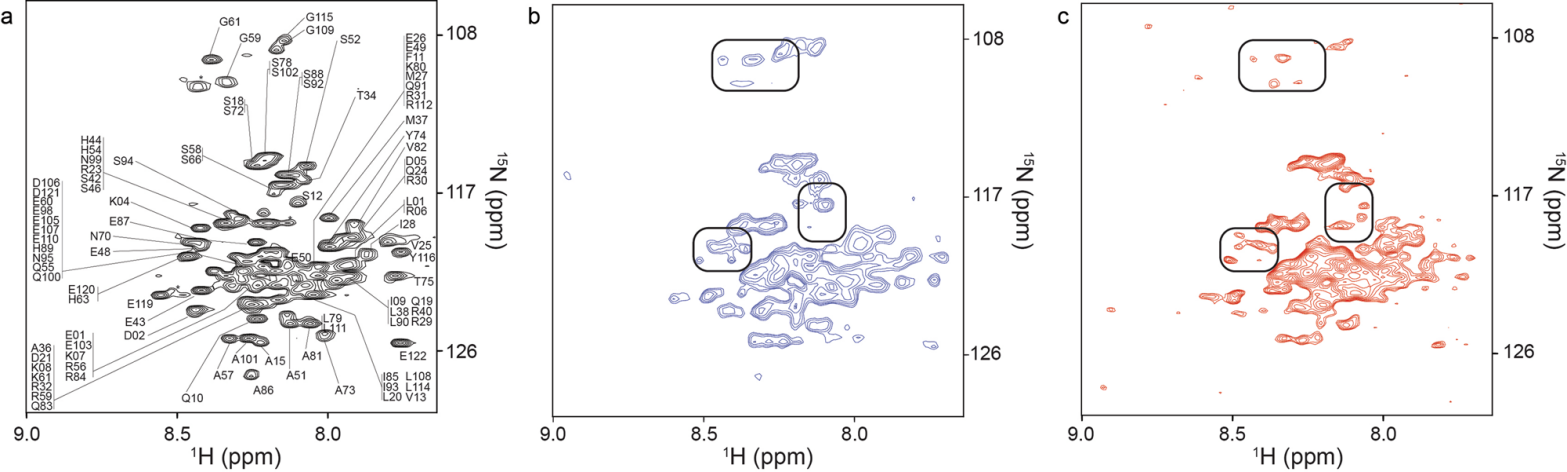
VECT-transfected cells portray the physiological state. **a** In vitro ^1^H-^15^N CRINEPT-HMQC-TROSY spectrum of [*U*- ^2^D, ^15^N]-DARPP-32_1-122_. **b** In-cell ^1^H-^15^N CRINEPT-HMQC-TROSY spectrum of VECT-transfected [*U*- ^2^D, ^15^N]-DARPP-32_1-122_. **c** In-cell ^1^H-^15^N CRINEPT-HMQC-TROSY spectrum of electroporation-transfected [*U*- ^2^D, ^15^N]-DARPP-32_1-122_. Boxed regions highlight differences in ^1^H-^15^N cross peak resolution between electroporated and VECT-transfected HeLa cells.

Previous characterization utilized DARPP-32_1-122_ constructs from rats; in this work a human DARPP-32_1-122_ construct was used (Supplementary Fig. 1). 103 out of 108 possible ^1^H-^15^N cross-peaks were assigned for the human construct in the buffer used for this study (Fig. 3a). HeLa cells were chosen to minimize specific interactions that affect the localization and activity of DARPP-32_1-122_ in neuronal cells.

DARPP-32 is known to engage in an extensive interaction network that results in the formation of complexes with molecular weights that exceed the detectability limit, ~50 kDa, when using pulse programs typically employed for in vitro work^39^. Indeed, the ^1^H-^15^N heteronuclear single quantum coherence, HSQC, NMR spectrum of HeLa cells electroporated with [*U-* ^15^N] DARPP-32_1-122_ resulted in no interpretable cross-peaks (Supplementary Fig. 2). Perdeuterating target proteins and collecting cross-relaxation-enhanced polarization transfer heteronuclear multiple quantum coherence transverse relaxation-optimized, ^1^H-^15^N CRINEPT-HMQC-TROSY, NMR spectra on in-cell samples can circumvent this problem by facilitating detection of high molecular weight complexes^12^. Uniformly labeled [*U*- ^2^D, ^15^N]-DARPP-32_1-122_ was delivered to HeLa cells using VECT and electroporation. ^1^H-^15^N CRINEPT-HMQC-TROSY experiments affirmed that perdeuteration was required to obtain an in-cell spectrum of DARPP-32_1-122_ (Fig. 3b, c). The narrow chemical shift dispersion showed that the protein remained predominantly unfolded in-cell, with many of the in-cell cross-peaks lying very close to those observed in vitro (Fig. 3a). The spectra were consistent with intermediate exchange implying that DARPP-32_1-122_ may engage in transient quinary interactions that will result in cross-peak broadening.

The spectrum of electroporated cells (Fig. 3c) contained sharp cross-peaks not observed in cells containing VECT-transfected target protein (Fig. 3b). Unlike the case of VECT protein delivery, control experiments examining the supernatant of electroporated samples revealed sharp ^1^H-^15^N cross-peaks consistent with leakage of labeled target protein from the cells (Supplementary Fig. 3). This likely reflects the loss of integrity of plasma and nuclear membranes and other organelles due to the electroporation process^19,27,28^. The combination of prolonged cell viability and the absence of cell leakage suggests that VECT is a simple and reliable method to deliver exogenous target proteins for long-duration in-cell NMR studies. It should be noted that electroporation parameters, as well as those of other delivery methods, can be optimized to minimize cell damage, and viable cells can be isolated, although this procedure requires several additional hours^17^.

### DARPP-32 phosphorylation is not regulated in HeLa cells

In neuronal cells, the intracellular localization and activity of DARPP-32 is regulated by phosphorylation and dephosphorylation at several residues (Supplementary Fig. 1). Phosphorylation by cAMP-dependent protein kinase A, PKA, of residue T34 converts DARPP-32 into a potent inhibitor of protein phosphatase-1, PP1. As a PP1 inhibitor, DARPP-32 amplifies the activity of PKA at the plasma membrane and in the cytoplasm affecting a broad spectrum of potential targets and downstream functions and is a key target in combating neurological diseases^40–42^ and carcinogenesis^43^. Conversely, when phosphorylated at T75 by cyclin-dependent kinase 5, CDK5, DARPP-32 inhibits PKA signaling, abating inhibition of PP1^44^.^45^^,^ Amplification of PKA activity also results in the phosphorylation of protein phosphatase 2 A, PP-2A, and subsequent dephosphorylation at T75^46^. In the cytosol, where DARPP-32 predominates, S45 and S97 (S102 in humans) are phosphorylated by casein kinase 2, CK2, and require dephosphorylation at S97 (S102) for nuclear co-localization^32,34^. CK2-mediated phosphorylation enhances phosphorylation of T34 by PKA^47^ but the functional consequences of this interaction remain unresolved^44^. Thus the state of phosphorylation determines the cellular location and consequent activity of DARPP-32.

Antibodies that recognize phosphorylated T34, T75, and S102 were used to look for evidence of biochemical modification. Western blots of cell lysates revealed weak phosphorylation at S102, indicative of constitutively expressed and active CK2^48^. The extent of phosphorylation was not quantified. No phosphorylation at T34 or T75 was detected (Supplementary Fig. 4). The lack of T34 phosphorylation by cAMP-dependent PKA, which exists as an inactive tetramer, may be due to the absence of induction and/or the intracellular localization of PKA^49^. Regulation of cAMP/PKA signaling is controlled by A-Kinase Anchor proteins, AKAPs, which confine PKA to subcellular compartments close to its targets, thus limiting its activity^50^. The lack of phosphorylation of T75 by CDK5 is likely due to the absence of regulatory neuronal activators p35, p39, and cyclin-I in HeLa cells^51^.

VECT-transfected cells were treated with 10 µM Forskolin, a small drug-like molecule, which activates adenylyl cyclase and downstream cAMP-sensitive enzymes such as PKA, altering metabolic fluxes in the cell. Western blots indicated no change in the extent of S102 phosphorylation after Forskolin treatment (Supplementary Fig. 4). The absence of changes suggests that the regulation of DARPP-32 activity may be cell-specific. Indeed, it is not known the extent to which DARPP-32 is expressed in HeLa cells^52^, so it is not surprising to suspect that many of the regulatory elements are not present at the required concentrations or intracellular locations. It is also possible that elements from the C-terminal half of the molecule or intact DARPP-32 is required for full regulation of phosphorylation and dephosphorylation activity.

**Fig. 4:**
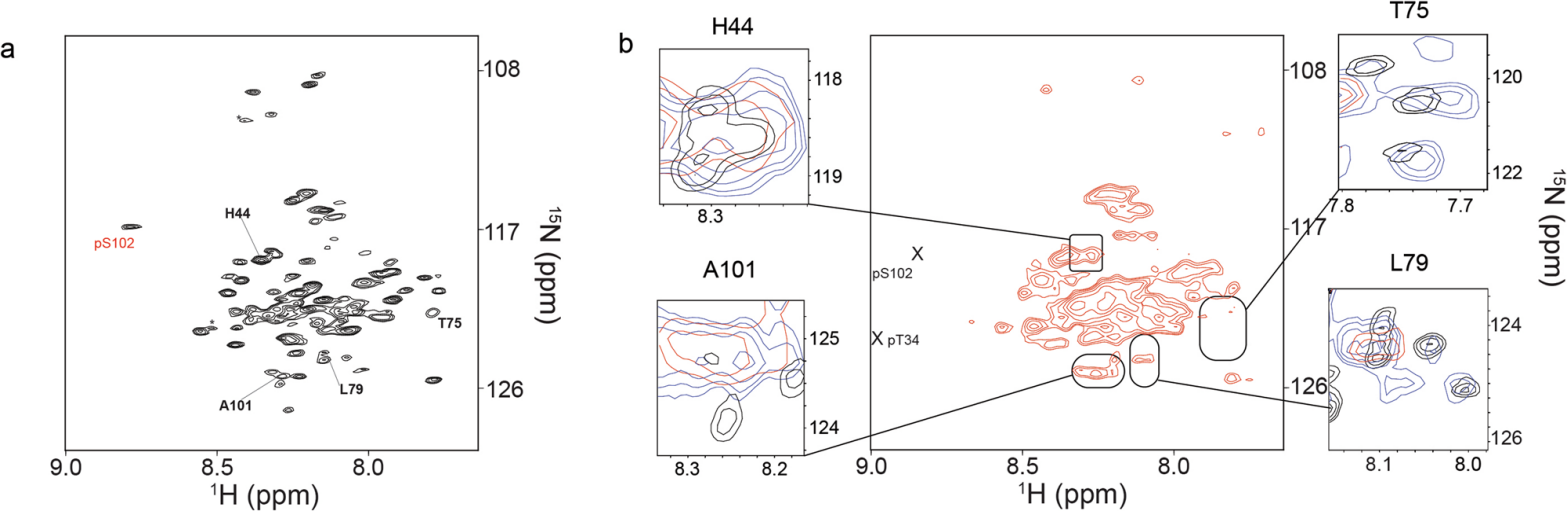
Forskolin treatment results in broadening of the DARPP-32_1-122_ in-cell NMR spectrum. **a** In vitro ^1^H-^15^N HSQC spectrum of [*U*- ^15^N]-DARPP-32_1-122_ treated with CK2. The cross-peak associated with phosphorylated S102 is in red. Bold residues are the same as in the insets on the right. **b** In-cell ^1^H-^15^N CRINEPT-HMQC-TROSY spectrum of [*U*- ^2^D, ^15^N]-DARPP-32_1-122_ treated with Forskolin. Boxed regions show overlays from the in vitro ^1^H-^15^N HSQC spectrum of [*U*- ^15^N]-DARPP-32_1-122_ (black) and the in-cell ^1^H-^15^N CRINEPT-HMQC-TROSY spectrum of [*U*- ^2^D, ^15^N]-DARPP-32_1-122_ acquired in the absence (blue) and presence (red) of Forskolin.

Purified [*U*- ^15^N]-DARPP-32_1-122_ was treated in vitro with PKA, which phosphorylates T34, and CK2, which phosphorylates S102. ^1^H-^15^N HSQC spectra were assigned to account for chemical shift changes associated with the biochemical modifications and to help identify modified ^1^H-^15^N cross-peaks in in-cell spectra (Supplementary Fig. 5). ^1^H-^15^N cross-peaks corresponding to phosphorylated T34 and S102 were observed in the ^1^H-^15^N HSQC spectra (Supplementary Fig. 5, Fig. 4a) but not in the in-cell ^1^H-^15^N CRINEPT-HMQC-TROSY spectrum of [*U*- ^2^D, ^15^N]-DARPP-32_1-122_ treated with Forskolin (Fig. 4b). This is not surprising since phosphorylation of T34 was not detected by Western blot analysis (Supplementary Fig. 4) and phosphorylation of S102 was sub-stoichiometric in HeLa cells.

The in-cell spectrum of [*U*- ^2^D, ^15^N]-DARPP-32_1-122_ treated with Forskolin is more extensively broadened than in untreated cells (Fig. 4b). Overlays of selected ^1^H-^15^N cross-peaks obtained in vitro and in-cell in the absence and presence of Forskolin reveal differential changes in chemical shifts and intensities (Fig. 4b boxed regions) often indicative of changes in quinary structure. The relevance of these changes was assessed by comparing the normalized cross-peak intensity ratios of [*U*- ^2^D, ^15^N]-DARPP-32_1-122_ from two biological replicate samples of VECT-transfected cells (Supplementary Fig. 6). About 90% of the cross-peak ratios were between 0.5 and 2 indicating a roughly two-fold variation in intensity (Supplementary Fig. 6). This level of biological variation, i.e., noise, precluded an accurate assessment of the location and extent of quinary structure changes due to Forskolin. Control experiments demonstrated that VECT-transfected cells maintained viability for at least the 90 min window required to prepare them for NMR spectroscopy. Beyond that, reduced intracellular oxygen and ATP were likely to cause the health of the cells to decline during the time required to collect the data, which may contribute to the spectral changes.

## Discussion

Microfluidics-based delivery of target protein into HeLa cells was tested and found to be a simple reliable method that preserves cell physiology for long-term in-cell NMR experiments. Direct comparison of protein delivery into cells showed that electroporation may deliver up to four times more protein under the same conditions. However, unlike electroporation and other pore-forming delivery techniques, which result in cell mortality within 24–48 h and can rupture internal membranous structures^19–21^, VECT-transfection does not require extensive optimization to effectively deliver target protein to 10^7^ eukaryotic cells and can be applied to most cell types and target proteins. The procedure can be accomplished in 20 min by using a standard syringe pump connected to a microfluidics chip (Fig. 1). Processed cells are uniformly loaded with protein and require minimal recovery time. Growth resumes immediately and is comparable to that of control cells (Fig. 2). The applicability of VECT delivery for drug screening and monitoring changes in physiology was demonstrated by collecting in-cell spectra in response to exogenous administration of Forskolin.

## Methods

### Chemicals and reagents

All chemicals used were of molecular biology grade or better.

### Plasmid construction

Synthetic DNA encoding for the 13 kDa N-terminal human DARPP-32 fragment, DARPP-32_1-122_ was subcloned into pET-28a(+) (Novagen) using *NdeI* and *XhoI* restriction sites (Genscript). The resulting plasmid, pET-28trDARPP-32 confers kanamycin resistance and expresses N-terminally 6XHis-tagged DARPP-32_1-122_ from the T7 lac promoter.

### Protein overexpression

Reduced proton density, REDPRO, uniformly labeled [*U-* ^2^D, ^15^N] DARPP-32_1-122_ was prepared as previously described^53^. Briefly, *E. coli* strain BL21(DE3) Codon+ was transformed with pET-28trDARPP-32 and 50 mL of Miller Lysogeny Broth (LB) containing 75 µg/mL of kanamycin was inoculated using a single colony and incubated overnight at 37 °C. The overnight culture was transferred into 1 L of fresh LB medium containing 75 µg/mL of kanamycin and allowed to grow at 37 °C to an OD_600_ of 0.7–0.9. Cells were centrifuged at 200 × *g* for 20 min at 37 °C and washed twice with minimal, M9, medium, and resuspended in 1 L of deuterated M9 medium containing 1 g/L of ^15^NH_4_Cl and 0.2% glucose as the sole nitrogen and carbon sources. The culture was incubated for 15–20 minutes at 37 °C to facilitate cell acclimation. Protein expression was induced by adding isopropyl β-d-1-thiogalactopyranoside to a final concentration of 1 mM and induction was allowed to proceed for 2–4 h. For experiments to assign backbone nuclei, ^13^C-glucose was used in place of the 0.2% glucose as the sole carbon source to prepare [*U-* ^15^N, ^13^C] DARPP-32_1-122_ and the final culture was overexpressed in non-deuterated M9 medium.

### Protein purification

DARPP-32_1-122_ was purified using a Ni-NTA column under denaturing conditions. Cells were resuspended in lysis buffer, 100 mM NaPO_4_, pH 8.0, 10 mM Tris, 8 M urea, and sonicated using a Model 250 Digital Sonifier (Branson) for seven cycles at 40% power using 0.3 s pulses and a 1.0 s rest between pulses for 60 total seconds of pulse time. The lysate was clarified by centrifugation at 30,000 × *g* for 45 min and loaded onto a column pre-equilibrated with lysis buffer. The column was washed with 50 mL of wash buffer, 100 mM NaPO_4_, pH 6.3, 10 mM Tris, 8 M urea, and the protein eluted with 20 mL of elution buffer, 100 mM NaPO_4_, pH 4.5, 10 mM Tris, 8 M urea. The eluent was dialyzed into buffer A, 50 mM NaPO_4_, pH 7.0, 50 mM NaCl, loaded onto a GE HiTrap^TM^ Q HP Column, and eluted with a 300 mL linear gradient from buffer A to buffer B, 50 mM NaPO_4_, pH 7.0, 1 M NaCl, using a Biorad DualFlow chromatography system (Biorad). The purified protein was exchanged into storage buffer, 10 mM sodium phosphate, pH 7.0, 100 mM NaCl, 0.01% sodium azide, and 20% glycerol, and concentrated to 100 mM by using an Amicon Ultra-15 Centricon (Millipore) for storage at −80 °C. Emerald GFP, EmGFP, was overexpressed from plasmid pRSET-EmGFP and purified as previously described^5^ and exchanged into the storage buffer.

### Protein transfection by electroporation

HeLa cells (Sigma) were prepared by seeding 4 × 10^6^ cells into five 15 cm Corning culture plates. Cells were incubated for 2–3 days in culture medium, Dulbecco’s Modified Eagle Medium, DMEM (Gibco), containing 4.5 g/L d-glucose, 110 mg/L sodium pyruvate, and supplemented with 10% fetal bovine serum, FBS (Gibco), to ~80% confluence (~12 × 10^6^ cells/plate). Cells were harvested as previously described^12^ by exposure to 0.25% trypsin EDTA (Gibco) for 3 min at 37 °C, pelleted by centrifugation at 200 × *g* for 6 min at 25 °C, washed twice with 5 mL of phosphate-buffered saline, PBS, and counted. Cells were resuspended in 100 µL of electroporation buffer, 100 mM NaPO_4_, pH 7.0, 5 mM KCl, 15 mM MgCl_2_, 15 mM HEPES, 5 mM ATP, 5 mM reduced glutathione and 50% Amaxa Nucleofector Solution R (Lonza, Inc). Purified [*U*- ^2^D, ^15^N] DARPP-32_1-122_ or GFP, in storage buffer, was added to a final concentration of 300 μM. Aliquots of 100 µL containing ~2 × 10^6^ cells were transferred into 1 mm cuvettes (Lonza) and electroporation was performed using an Amaxa Nucleofector 2b apparatus (Lonza) set to the B-28 pulse program as previously described^12,17,30^. Each cuvette was pulsed twice, with gentle agitation between pulses. 1 mL of prewarmed culture medium was added to each cuvette immediately following the second pulse and the cell suspension was transferred into a 50 mL centrifuge tube and incubated at room temperature for 20 min to maximize transfection and facilitate cell recovery. Cells were centrifuged at 200 × *g* for 3 min at RT, and washed twice using 2 mL of culture medium to remove residual protein. Samples were prepared for in-cell NMR by resuspending transfected cells in 450 µL of culture medium and 50 µL of D_2_O, and transferring the suspension to a 5 mm NMR tube. For plate reading to determine GFP concentrations, aliquots of 2 × 10^6^ cells were resuspended in 1 mL of RIPA buffer, 25 mM Tris-HCl, pH 7.6, 150 mM NaCl, 1% NP-40, 1% sodium deoxycholate, 0.1% SDS, and frozen at −80 °C.

### Protein transfection by microfluidics

HeLa cells (Sigma) were prepared by seeding 4 × 10^6^ cells into three 15 cm culture dishes (Corning). Seeded cells were incubated for 2–3 days in culture medium to ~80% confluence (~1.2 × 10^7^ cells/plate). Cells were harvested as described above^12^, passed through a 40 μm filter to reduce clumping, and counted. Approximately 3 × 10^7^ cells were resuspended in 3 mL of cell flow buffer, 0.4% BSA, 0.04% EDTA, 20% Percoll, 5 µL of Tween-20, and purified [*U*- ^2^D, ^15^N] DARPP-32_1-122_ or GFP, in storage buffer, was added to a final concentration of 300 µM. A three-channel polydimethylsiloxane, PDMS, Volume Exchange for Convective Transfer, VECT, device with rigid, 9.6-μm microchannels was prepared as previously described^22^. The VECT device was placed on a Vista Vision (VWR) microscope for observation and purged to remove trapped air and any existing manufacturing debris using passivation buffer, 1% BSA in phosphate-buffered saline (Gibco). The cell suspension was transferred to a 3 mL syringe, connected to a New Era Pump Systems Model 300 syringe pump set to a flow rate of 100 µL/min, and the flow commenced while observing for bubbles and blockages. The cell suspension eluent was collected and allowed to equilibrate for 20 min at room temperature to maximize protein transfection and facilitate cell recovery. Cells were centrifuged at 200 × *g* for 6 min at 25 °C and washed twice with 5 mL of PBS. Samples were prepared for in-cell NMR by resuspending transfected cells in 450 µL of culture medium with or without 10 µM Forskolin, and 50 µL of D_2_O, and transferring the suspension to a 5 mm NMR tube. For plate reading to determine GFP concentrations, aliquots of 2 × 10^6^ cells were resuspended in 1 mL of RIPA buffer, and frozen at −80 °C. About 5 × 10^6^ cells were reserved for plate reading as described above. Following VECT delivery of DARPP-32_1-122_, ~5 × 10^6^ cells were resuspended in 1 mL of RIPA buffer and frozen at −80 °C in preparation for future Western blotting.

### NMR experiments

All NMR spectra were recorded at 298 K on either a 700 MHz Avance II NMR spectrometer (Brüker) equipped with a TXI cryoprobe or a 600 MHz Avance III NMR spectrometer equipped with a QCI-P cryoprobe. All in vitro samples were prepared by combining 450 µL of purified labeled DARPP-32_1-122_ in NMR buffer, 50 mM NaPO_4_, pH 6.8, with 50 µL of D_2_O, to a final concentration of 100 μM. In-cell samples were prepared by combining 450 µL of cells suspended in a culture medium and 50 µL of D_2_O. Spectra were processed with Topspin (version 3.2, Brüker) and analyzed using CARA software^54^.

Heteronuclear single quantum coherence, ^1^H-^15^N HSQC, experiments were performed with Watergate water suppression^55^ and the spectra were acquired with 64 transients and 1024 and 128 points in the ^1^H and ^15^N dimensions, respectively. The spectral widths in the ^1^H and ^15^N dimensions were 14 and 35 ppm, respectively.

Cross-relaxation-enhanced polarization transfer heteronuclear multiple quantum coherence transverse relaxation-optimized, ^1^H-^15^N CRINEPT-HMQC-TROSY^56^, experiments were performed with Watergate water suppression using CRIPT transfer delays of 1.5 ms and a recycle time of 300 ms. 512 transients were used to acquire 1024 and 128 points with spectral widths of 14 and 35 ppm in the proton and nitrogen dimensions, respectively.

A standard array of triple resonance experiments, NCACB, CBCACONH, HNCA, HNCO, HNCOCA, HNCACO, (H)CC(CO)NH, and H(CC)(CO)NH^39^, were used to assign backbone nuclei of both unphosphorylated and phosphorylated [*U-* ^15^N, ^13^C] DARPP-32_1-122_. Assignments were accomplished using CARA software.

Biological replicate HSQC experiments were performed after transfecting DARPP-32_1-122_ into HeLa cells using VECT. CARA software was used to obtain the intensity values for each experiment. The intensities were normalized using a glutamine amide side chain at 7.49 ppm and 112.4 ppm in the proton and nitrogen dimensions, respectively, that did not undergo changes in chemical shift. The errors in the ratios were derived by propagating the errors in the individual cross-peak intensities_._ All experiments were performed independently at least twice and the results were combined for analysis by using the ANOVA statistical package in Prism 6.0 (Graphpad, Inc).

### Phosphorylation of DARPP-32_1-122_

Phosphorylation of DARPP-32_1-122_ was performed as previously described^35^. Purified [*U*- ^2^D, ^15^N] DARPP-32_1-122_ was exchanged into NE Buffer (New England Biolabs) 50 mM Tris-HCl, pH 7.5, 10 mM MgCl_2_, 0.1 mM EDTA, 2 mM DTT, 0.01% Brij 35, and 200 µM ATP. 8 µg/mL of protein kinase A, PKA (New England Biolabs), or casein kinase 2, CK2 (New England Biolabs), was added to initiate the reaction. Each reaction was allowed to proceed at 30 °C for 90 min. The phosphorylated protein solution was immediately combined into 450 µL of NMR buffer and 50 µL of D_2_O for NMR spectroscopy, or 1:1 with Laemmli buffer for SDS-PAGE and Western blotting. SDS-PAGE band intensities were measured by using ImageJ software.

### Forskolin treatment of HeLa cells

Aliquots containing ~5 × 10^6^ HeLa cells that had undergone VECT delivery of [*U*- ^2^D, ^15^N] DARPP-32_1-122_ were suspended in 1 mL of culture medium. The cells were treated with 10 µM Forskolin (TCI) and incubated at 37 °C for 30 min. The cells were centrifuged at 200 × *g* for 6 min at 25 °C, resuspended in 1 mL of RIPA buffer, and stored at −80 °C for SDS-PAGE and Western blotting.

### Cell viability and morphology assays

Cells were collected at the end of the 20 min rest period following protein delivery to measure the initial survival rates and viability of VECT- and electroporation-transfected cells. Individual samples were combined into a 50 mL conical tube (Thermo), washed twice with 10 mL of PBS (Gibco) to remove residual protein, and resuspended in 2 mL of culture medium in duplicate. To assess initial survival rates in the critical 90 min window where cells are prepared for in-cell NMR, a 10 µL aliquot of cell suspension was removed every 10 min, diluted 1:10 (v/v) with 0.4% Trypan blue (Thermo Fisher) and counted with a hemocytometer (Reichert). To assess long-term viability (48 h), eight 35 mm tissue culture dishes (Corning) were individually seeded with 0.3 × 10^6^ cells across three conditions (electroporation-, VECT- and non-transfected cells). Pairs of dishes for each condition were harvested and counted at 12 h intervals. Cell images were taken using an Evos^®^ FL auto imaging system (Thermo Fisher Scientific) 12 h after protein delivery to assess morphology and fluorescence.

### Western blotting

HeLa cell samples from electroporation-, and VECT-transfections, ±Forskolin, were individually thawed and lysed using a Model 250 Digital Sonifier (Branson). The lysate was centrifuged at 200 × *g* for 30 min to pelletize cellular debris and the supernatant was decanted. A 1:1 dilution of the clarified lysate was prepared for electrophoresis using 2x Laemmli buffer. The same procedure was followed to create a control sample using ~5 × 10^6^ HeLa cells that had not undergone transfection. Whole-cell lysates and samples from in vitro phosphorylation reactions were subjected to SDS-PAGE (Mini-PROTEAN Tetra Cell, Bio-Rad). Protein transfer to a 0.2 µm nitrocellulose membrane (Biorad) was performed at 40 V for 16 h. Four membranes were blotted using recombinant anti-DARPP-32 rabbit antibody (EP720Y/AB40801, 1:1000 dilution, Abcam), phospho-DARPP-32 (Ser97 in rat or S102 in human) rabbit monoclonal antibody (D11A5, 1:1000 dilution, Cell Signaling Technology), phospho-DARPP-32 (T34) rabbit monoclonal antibody (D27A4, 1:1000 dilution, Cell Signaling Technology), and phospho-DARPP-32 (T75) rabbit polyclonal antibody (AB51114, 1:3000 dilution, Abcam). Anti-DARPP-32 was used to initially determine the effectiveness of the blotting protocol. Chemiluminescence was generated using ECL western blotting substrate (Promega) and detection and imaging was performed by using a ChemiDoc^TM^ MP imaging system (Bio-Rad).

### Fluorometric quantitation of intracellular GFP concentrations

Duplicate sets of six samples of ~2 × 10^6^ cells each from the electroporation and microfluidics transfections, along with a control sample of ~2 × 10^6^ non-transfected HeLa cells, were thawed and lysed using a Model 250 Digital Sonifier (Branson). Each of the samples were centrifuged at 200 × *g* for 30 min at room temperature to pelletize cellular debris and the supernatant was collected. About 150 µL of each of the transfected, control, and background samples were transferred into a 96-well plate (Model 3603, Costar). Fluorescence was detected using a Synergy HT plate reader (BioTek). A calibration curve was generated using purified GFP. To calculate the final concentration of GFP delivered per cell, a HeLa cell volume of 2500 µm^3^ was assumed.

### Reporting summary

Further information on research design is available in the Nature Research Reporting Summary linked to this article.

## Supplementary information

Supplementary Information

Description of Additional Supplementary Files

Supplementary Data 1

Reporting Summary

## Data Availability

All data are available upon request. The source data behind the graphs and uncropped and unedited gel images are included in Supplementary Data 1 and Supplementary Fig. 7, respectively.

## Materials availability

All materials are available upon request.
