# Author Correction: Microfluidics delivery of DARPP-32 into HeLa cells maintains viability for in-cell NMR spectroscopy

**DOI:** 10.1038/s42003-022-03649-6

**Published:** 2022-07-07

**Authors:** Nicholas Sciolino, Anna Liu, Leonard Breindel, David S. Burz, Todd Sulchek, Alexander Shekhtman

**Affiliations:** 1 University at Albany, Department of Chemistry, Albany, NY 12222 USA; 2 Georgia Tech, School of Mechanical Engineering, Atlanta, GA 30332 USA

**Keywords:** Solution-state NMR, Intracellular signalling peptides and proteins, Molecular neuroscience, Solution-state NMR

Correction to: *Communications Biology* 10.1038/s42003-022-03412-x, published online 12 May 2022.

In the original version of the Article, the Acknowledgements section was not shown. The Acknowledgements should read:

**Acknowledgements**

The project described was supported by Award Numbers 1P01HL146367-01 and R01GM085006 from the U.S. National Institute of Health. The content is solely the responsibility of the authors and does not necessarily represent the official view of the NIH.

This has now been corrected in the PDF and HTML versions of the Article.

