# Supplementary material for: Microfluidics delivery of DARPP-32 into HeLa cells maintains viability for in-cell NMR spectroscopy
Source: Commun Biol. 2022 May 12;5:451. doi: 10.1038/s42003-022-03412-x (PMC9098904; doi:10.1038/s42003-022-03412-x)
Supplement: Supplementary file 1 — Supplementary Information [file 42003_2022_3412_MOESM1_ESM.pdf]

Supplementary Information

Microfluidics delivery of DARPP32 into HeLa cells maintains viability for in-cell NMR spectroscopy.

Nicholas Sciolino[1], Anna Liu[2], Leonard Breindel[1], David S. Burz[1], Todd Sulchek[2], Alexander Shekhtman[1*]

[1]University at Albany, Department of Chemistry, Albany, NY 12222
[2]Georgia Tech, School of Mechanical Engineering, Atlanta, GA30332

*Corresponding author; email: ashekhtman@albany.edu

Supplementary Information

List of Supplementary Materials:

Supplementary Figure 1. Protein sequence homology between human, rat and mouse DARPP-$32_{1\text{-}122}$.

Supplementary Figure 2. ^{1}H-^{15}N HSQC, NMR spectrum of HeLa cells electroporated with [*U*- ^{15}N] DARPP-$32_{1\text{-}122}$.

Supplementary Figure 3. ^{1}H-^{15}N HSQC NMR spectra of HeLa cell supernatants following in-cell NMR spectroscopy.

Supplementary Figure 4. Western blots of DARPP-$32_{1\text{-}122}$ phosphorylation.

Supplementary Figure 5. ^{1}H-^{15}N HSQC spectra of [*U*- ^{15}N]-DARPP-$32_{1\text{-}122}$ phosphorylated *in vitro*.

Supplementary Figure 6. Reproducibility of in-cell ^{1}H-^{15}N CRINEPT-HMQC-TROSY spectra of VECT-delivered [*U*- ^{2}D, ^{15}N]-DARPP$32_{1\text{-}122}$.

Supplementary Figure 7. Uncropped Image for Supplementary Figure 4

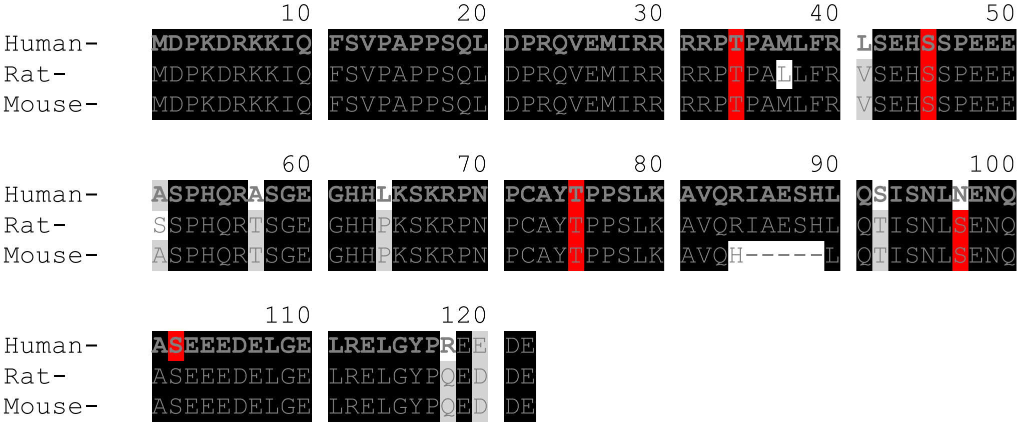

Supplementary Figure 1. Protein sequence homology between human, rat and mouse DARPP-32$_{1\text{-}122}$. Black highlights indicate identical residues in all species, grey highlights indicate identical or similar residues between two of the species. Red highlights primary sites of phosphorylation.

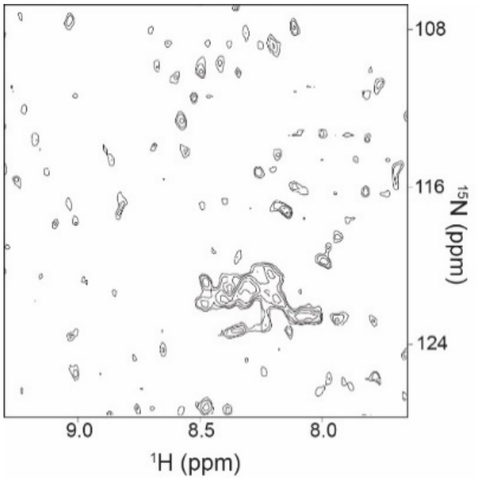

Supplementary Figure 2. ^{1}H-^{15}N HSQC NMR spectrum of HeLa cells electroporated with [*U*- ^{15}N] DARPP-$32_{1\text{-}122}$.

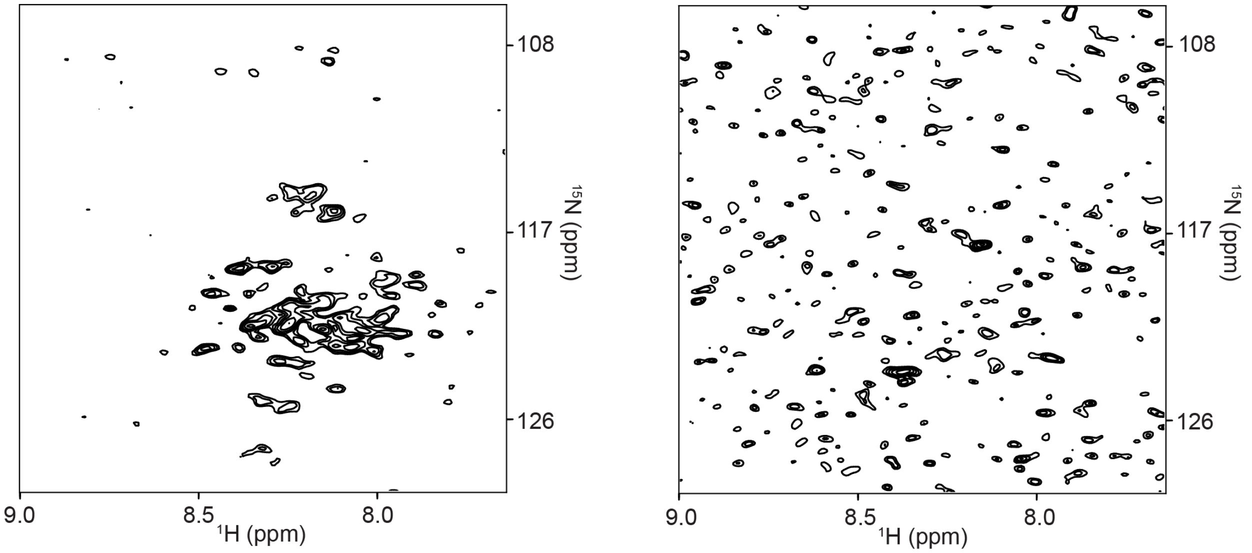

Supplementary Figure 3. ^{1}H-^{15}N HSQC NMR spectra of HeLa cell supernatants following in-cell NMR spectroscopy. HeLa cells were transfected with [*U*- ^{15}N] DARPP-$32_{1\text{-}122}$ by using electroporation (left) and VECT (right). The sharp peaks in the electroporated cells are evidence of leakage.

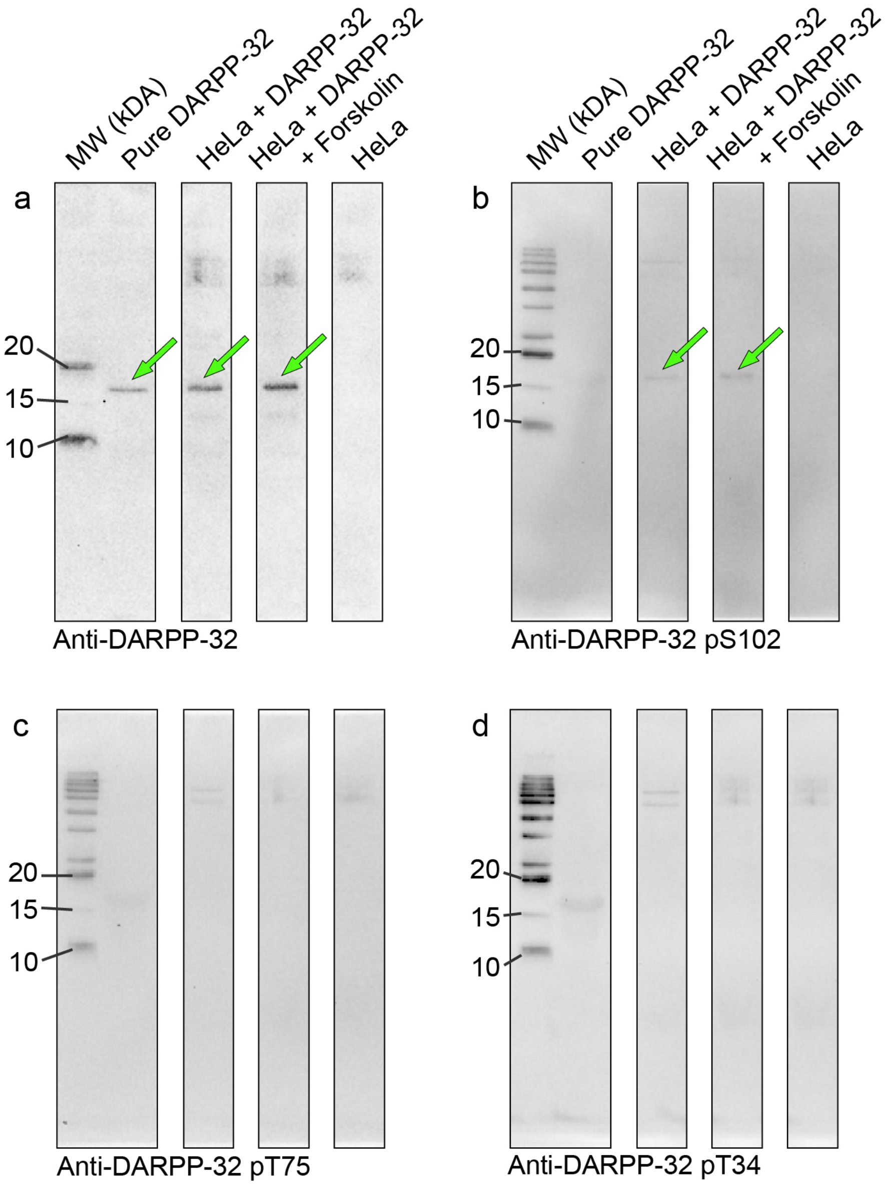

Supplementary Figure 4. Western blots of DARPP-$32_{1\text{-}122}$ phosphorylation. a). Western blot of DARPP-$32_{1\text{-}122}$ following VECT transfection confirming protein delivery into HeLa cells. b) Western blot of phospho-S102 indicates the presence of phosphorylated S102 before and after treatment with Forskolin. c) Western blot of phospho-T75 indicates the absence of phosphorylated T75 before and after treatment with Forskolin. d) Western blot of phospho-T34 indicates the absence of phosphorylated T34 before and after treatment with Forskolin.

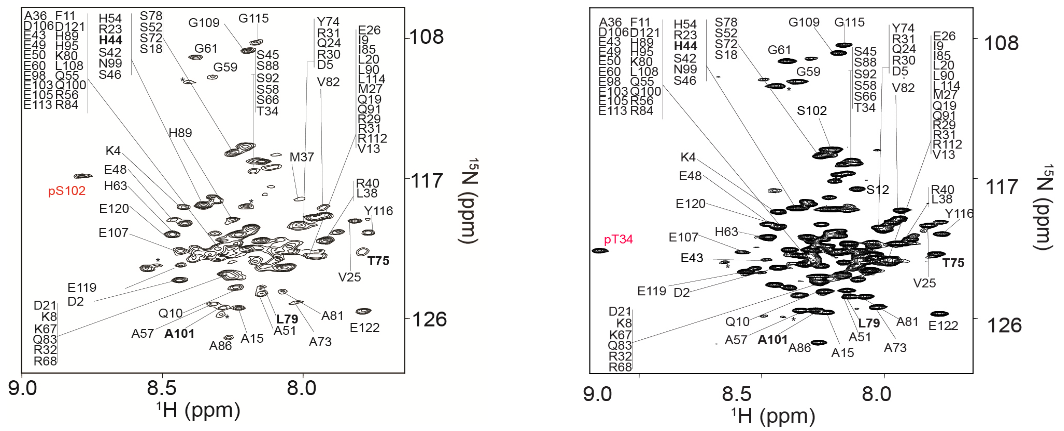

Supplementary Figure 5. ^{1}H-^{15}N HSQC spectra of [*U*- ^{15}N]-DARPP-$32_{1\text{-}122}$ phosphorylated *in vitro*. (Left) treated with CK2. (Right) treated with PKA. Assignments are shown for phosphorylated species. ^{1}H-^{15}N cross peaks corresponding to phosphorylated S102 and T34 are in red.

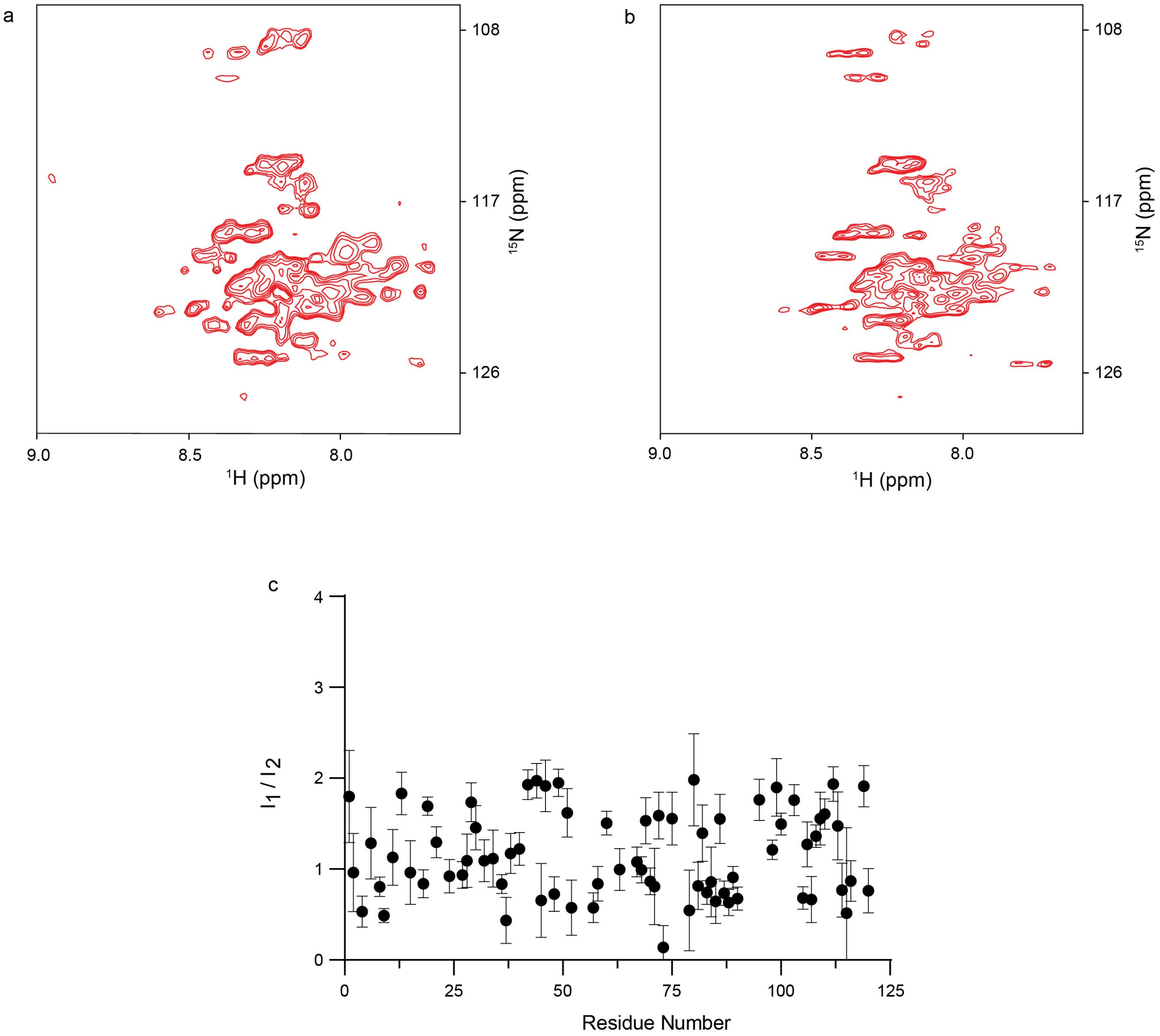

Supplementary Figure 6. Reproducibility of in-cell ^{1}H-^{15}N CRINEPT-HMQC-TROSY spectra of VECT-delivered [*U*- ^{2}D, ^{15}N]-DARPP32$_{1\text{-}122}$. Spectra a and b were obtained from separately cultured cells. C. Intensity ratios of DARPP32$_{1\text{-}122}$ cross peaks. Most intensity ratios were between 0.5 and 2 (red dashed lines). showing the average scatter in these experiments. The scatterplot was generated using Prism (GraphPad).

a

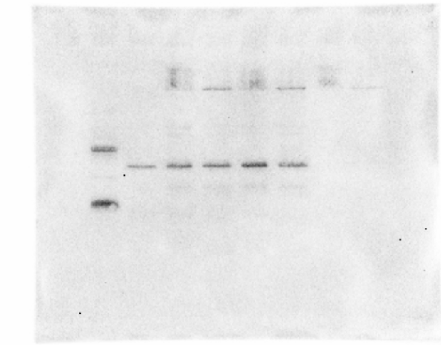

Anti-DARPP-32

b

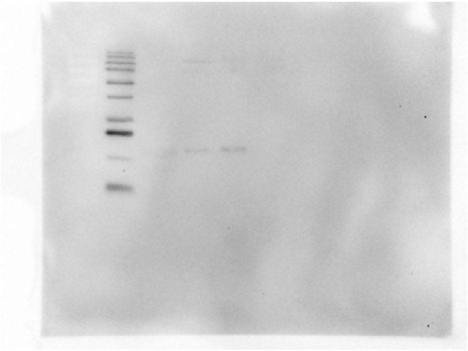

Anti-DARPP-32 pS102

c

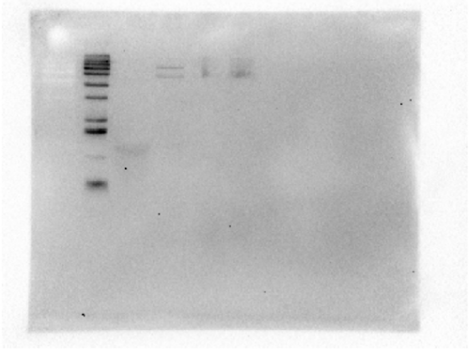

Anti-DARPP-32 pT75

d

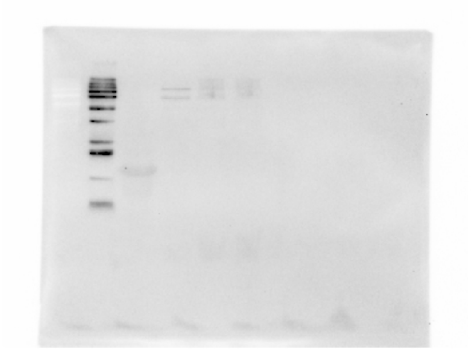

Anti-DARPP-32 pT34

Supplementary Figure 7 Uncropped Images for Supplementary Figure 4.
